# Polymyxin B Conjugates with Bio-Inspired Synthetic Polymers of Different Nature

**DOI:** 10.3390/ijms24031832

**Published:** 2023-01-17

**Authors:** Anna Dvoretckaia, Tatiana Egorova, Apollinariia Dzhuzha, Mariia Levit, Eugene Sivtsov, Elena Demyanova, Evgenia Korzhikova-Vlakh

**Affiliations:** 1 Institute of Chemistry, Saint-Petersburg State University, 198504 St. Petersburg, Russia; 2 Institute of Macromolecular Compounds, Russian Academy of Sciences, 199004 St. Petersburg, Russia; 3 State Research Institute of Highly Pure Biopreparations FMBA of Russia, 197110 St. Petersburg, Russia; 4 Department of Physical Chemistry, Saint-Petersburg State Institute of Technology (Technical University), 190013 St. Petersburg, Russia

**Keywords:** polymyxin B, polymer conjugates, polypeptides, glycopolymer, prolonged antibiotic release, antimicrobial activity

## Abstract

The emergence and growth of bacterial resistance to antibiotics poses an enormous threat to humanity in the future. In this regard, the discovery of new antibiotics and the improvement of existing ones is a priority task. In this study, we proposed the synthesis of new polymeric conjugates of polymyxin B, which is a clinically approved but limited-use peptide antibiotic. In particular, three carboxylate-bearing polymers and one synthetic glycopolymer were selected for conjugation with polymyxin B (PMX B), namely, poly(α,L-glutamic acid) (PGlu), copolymer of L-glutamic acid and L-phenylalanine (P(Glu-*co*-Phe)), copolymer of N-vinyl succinamic acid and N-vinylsuccinimide (P(VSAA-*co*-VSI)), and poly(2-deoxy-2-methacrylamido-D-glucose) (PMAG). Unlike PGlu and PMAG, P(Glu-*co*-Phe) and P(VSAA-*co*-VSI) are amphiphilic and form nanoparticles in aqueous media. A number of conjugates with different polymyxin B loading were synthesized and characterized. In addition, the complex conjugates of PGLu or PMAG with polymyxin B and deferoxamine (siderophore) were obtained. A release of PMX B from Schiff base and amide-linked polymer conjugates was studied in model buffer media with pH 7.4 and 5.8. In both cases, a more pronounced release was observed under slightly acidic conditions. The cytotoxicity of free polymers and PMX B as well as their conjugates was examined in human embryonic kidney cells (HEK 293T cell line). All conjugates demonstrated reduced cytotoxicity compared to the free antibiotic. Finally, the antimicrobial efficacy of the conjugates against *Pseudomonas aeruginosa* was determined and compared. The lowest values of minimum inhibitory concentrations (MIC) were observed for polymyxin B and polymyxin B/deferoxamine conjugated with PMAG. Among the polymers tested, PMAG appears to be the most promising carrier for delivery of PMX B in conjugated form due to the good preservation of the antimicrobial properties of PMX B and the ability of controlled drug release.

## 1. Introduction

The growing number of drug-resistant pathogenic bacteria poses a global threat to human health. Wide use of antibiotics, including wrong choice of antibacterial drugs, erroneous dosages and uncontrolled prophylactic actions, has led to the emergence of drug-resistant bacterial strains. According to the World Health Organization (WHO), increasing resistance to traditional antimicrobial drugs poses a global public health risk [1]. In this regard, the search for new antibiotics, among which antimicrobial peptides (AMPs) occupy a special place, is of great importance.

AMPs have a variety of structures (linear and cyclic peptides, glycopeptides and lipopeptides) and properties [2]. Many antimicrobial peptides have a rapid effect on the cell wall and outer membrane of bacteria, causing their destruction and/or disorganization [3]. Non-membranolytic mechanisms play also an important role, e.g., for inhibition of nucleic acid biosynthesis and metabolism or inhibition of protein folding. Often, the effect of AMPs on bacteria is complex and combines several mechanisms simultaneously. Because of the complexity of the AMP action, resistance to them occurs much less frequently [4]. Furthermore, AMP advantages include fast action and low minimum inhibitory concentrations (MIC). 

Despite the fact that a large number of AMPs are known, only a few of them are currently used in clinical practice. For example, only polymyxins (polymyxin B and E (colistin)) for Gram-negative bacteria, glycopeptides (vancomycin, oritavancin, dalbavancin, teicoplanin and telavancin), bacitracin and daptomycin for Gram-positive bacteria, and gramicidin, affecting both types, are approved by the Food and Drug Administration (FDA) agency [5,6,7]. 

It should be noted that in addition to the positive aspects of AMPs, their use has also a number of limitations [8]. First is their low stability in physiological fluids, especially in the presence of proteases. Another important drawback is the cytotoxic properties of AMPs, especially neuro- and nephrotoxicity, which are typical, for example, for antibiotics of the polymyxin (PMX) group. Despite the high efficacy of PMXs against Gram-negative bacteria, they have restricted application and are mostly used as local therapeutics or as “antibiotics of last choice” in combined therapy.

The development of AMP delivery systems can be a successful solution in overcoming existing obstacles in their application. In recent years, a number of delivery systems of different nature were reported [9,10,11,12,13,14,15,16]. Among them, there are systems physically retaining AMPs [9,10,11,12,13] and covalent conjugates [16,17,18]. For example, the preparation of nanoparticles (NPs) by complexation of colistin (PMX E) with poly(glutamic acid) stabilized by PEG-derivative [9], hyaluronan/colistin [10] or oligochitosan/starch/colistin/tobramycin polyelectrolyte complexes [14] were recently reported as improved PMX delivery systems. The use of delivery systems can offer prolonged plasma half-life, reduced toxicity and improved pharmacodynamic properties. 

Compared to noncovalent polymer/AMP complexes, from which AMP is easily displaced by another polyanion in vivo, covalent AMP conjugates with a biocompatible polymer are more stable delivery systems. At the same time, the presence of hydrolysable bonds can provide a gradual release of the antibiotic from the conjugate. Currently, the synthesis of polymyxin conjugates has become the subject of intensive research. In particular, the development of conjugates of colistin with poly(ethylene glycol) [19], poly(ethylene glycol acrylate) [20], dextrin [16,18] and hyaluronan [21], as well as PMX B with vancomycin [15], human immunoglobulin G [22], alginate oligosaccharides [17], and gold nanoparticles and quantum dots [23] have been reported. The release of PMX from the conjugates is proposed as a result of the action of α-amylase [18,24], esterase or alginate lyase activity [17], lysozyme [21] or non-enzymatic hydrolysis [20].

Among the attractive strategies for effective antibacterial therapy, the application of siderophores for targeted delivery of antibiotics to bacteria is one of the most promising approaches [25]. Delivery of antibiotics mediated by siderophores has been compared to the Trojan Horse strategy [26], which mainly targets antibiotic-resistant bacteria. Siderophores are small iron (III)-chelating molecules that are necessary for bacteria to acquire and solubilize Fe^3+^ ions from the host organism. Iron plays a tremendous role in the most vital enzymatic processes of many living (micro)organisms, including bacteria. To provide their life cycle with iron, both Gram-positive and Gram-negative bacteria secrete siderophores to the extracellular environment to capture and import iron [26,27]. The latter is ensured by the recognition of the iron–siderophore complex by highly specific receptor proteins of the bacterial cell wall and active transport across the membrane of bacterium. The use of siderophores as vectors can help overcome membrane-mediated resistance of bacteria that produce different protection mechanisms [26,27]. Currently, there are many studies devoted to conjugation of siderophore with a drug which is not able to cross the bacterial membrane for delivery into the bacterial cell [25,26,27].

In this study, several biocompatible and bio-inspired synthetic polymers of different nature containing moieties of natural molecules (amino acids, N-glucosamine or succinic acid) were tested as carriers for PMX B conjugation. In particular, three negatively charged polymers and a neutral glycopolymer were selected. Recently, copolymer of L-glutamic acid and L-phenylalanine (P(Glu-*co*-Phe)) [28] and poly(α,L-glutamic acid) (PGlu) [29] were tested as PMX B and E delivery systems, in which positively charged PMXs were bound to negatively charged polymers due to polyelectrolyte interactions. Here, PMX B was conjugated with P(Glu-*co*-Phe) and PGlu to compare the properties of physically loaded and covalently bound PMX B. In addition, two synthetic biocompatible polymers, namely poly(2-deoxy-2-methacrylamido-D-glucose) (PMAG) [30] and copolymer of vinyl succinamic acid and N-vinylsuccinimide (P(VSAA-*co*-VSI)) [31], were also investigated as carriers for PMX B conjugation. The composition of PMX B polymer conjugates was optimized in terms of conjugation efficacy and physicochemical characteristics of conjugates. In addition, the complex polymer conjugates of PMX B and siderophore were synthesized. For this purpose, clinically approved deferoxamine (DFOA) was selected as a model siderophore. Finally, the antimicrobial efficacy of the conjugates against *Pseudomonas aeruginosa* was determined and compared.

**Figure 1:**
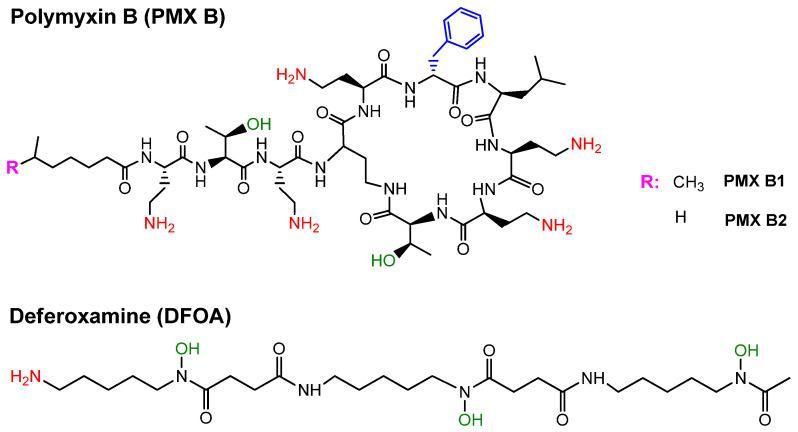
Structures of polymyxin B and deferoxamine used for conjugation with synthetic polymers. Polymyxin B used in this study was a mixture of homologues B1 (81.5%) and B2 (18.5%).

The chemical structures of functional targets used for conjugation with polymers are illustrated in Figure 1. The PMX B used consisted of two homologues, B1 and B2, which differed in the length of aliphatic tail. 

## 2. Results and Discussion

### 2.1. Polymer Carriers 

The structure and characteristics of polymers selected as carriers for PMX B are presented in Table 1. Two biodegradable polypeptides, namely, P(Glu-*co*-Phe) and PGlu, were synthesized by ring-opening polymerization of N-carboxyanhydrides of α-amino acids using a previously developed protocol [28]. Two other polymers represent the biocompatible macromolecules with non-biodegradable main chain. Both PMAG and PVSI were synthesized by reversible fragmentation-addition chain-transfer polymerization as described earlier [30,31]. Unlike water-soluble PMAG, PVSI is soluble in organic solvents. All used copolymers had number average molecular weight (*M_n_*) below 25,000 and narrow dispersity (*Ð*).

**Figure 2:**
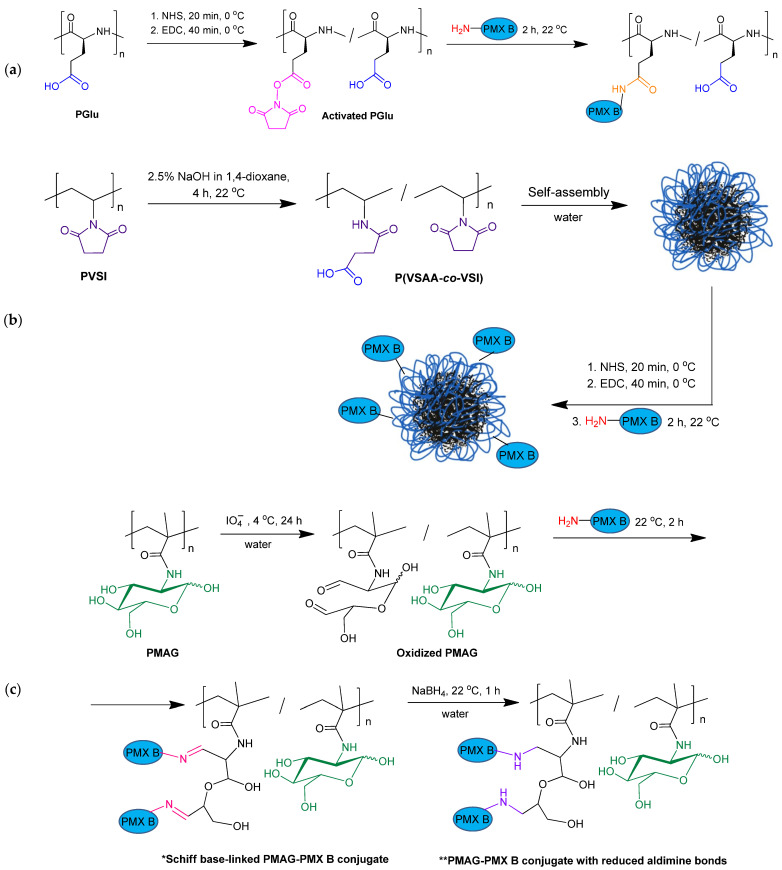
Scheme of modification of PGlu (**a**), PVSI (**b**) and PMAG (**c**), and conjugation with PMX B. For P(Glu-*co*-Phe), activation was performed as for PGlu, but the conjugate was in the form of modified nanoparticles as for P(VSAA-*co*-VSI).

As can be seen, P(Glu-*co*-Phe) and PGlu contain carboxylic groups suitable for conjugation after activation with amino-bearing compounds. Both PMAG and PVSI do not contain functional groups suitable for direct conjugation with PMX B and need to be modified to generate reactive functionality. In the case of PMAG, partial glucose oxidation was performed to generate aldehyde functionality highly reactive towards amino groups. For PVSI, the partial alkaline hydrolysis of succinimide ring to succinamic acid was selected to generate carboxyl groups. The scheme illustrating the conjugation of PMX to different (co)polymers is shown in Figure 2. The details on modification and conjugation of PMX B to these (co)polymers are discussed below (Section 2.3 and Section 2.4).

### 2.2. Synthesis and Characterization of Conjugates Based on Polypeptides 

The reaction between primary amino groups of PMX B and activated ester groups of glutamic acid in (co)polymer was utilized for conjugation of PMX B with P(Glu-*co*-Phe) or PGlu. Activation of carboxylic groups was performed in water with the use of water-soluble carbodiimide and N-hydroxysuccinimide (Figure 1). It is worth noting that the amphiphilic P(Glu-*co*-Phe) is self-assembled and forms nanoparticles in water. Thus, the activation of carboxylic groups and conjugation of PMX B with this polymer occurs on the surface of nanoparticles. 

Table 2 summarizes the amounts of PMX B used for conjugation and the conjugation efficacy. Taking into account that PMX B is a sterically massive substituent (*M_r_* = 1189), the activation density was set as 10 mol% of the total glutamic acid content in the copolymer. The amount of PMX B taken for the modification ranged from 3 to 7 mol% with respect to the carboxyl groups of the polymer. After synthesis, to avoid PMX retention by the polymer due to polyelectrolyte interactions, the unbound PMX was removed by ultrafiltration with the saline solution. The conjugation was testified by ^1^H NMR spectroscopy (Figure S1, Supplementary Materials). Due to the large number of overlapping signals for the free and conjugated PMX B, the calculation of the conjugated peptide antibiotic from the ^1^H NMR spectra was not possible. However, the appearance in the polymer spectrum of signals at 0.25–1.50 ppm, characteristic to PMX B, qualitatively indicates that the conjugation was successful. The amount of conjugated PMX B was calculated as the difference between the initial and unbound PMX amounts determined by ion-exchange HPLC (Figure S2, Supplementary Materials).

**Figure 3:**
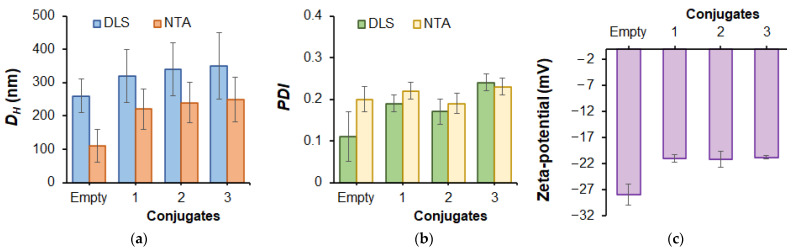
Characteristics of self-assembled empty P(Glu-*co*-Phe) and PMX B-conjugated nanoparticles determined by dynamic light scattering, nanoparticle tracking analysis and electrophoretic light scattering: hydrodynamic diameter (*D_H_)* (**a**), polydispersity index (PDI) (**b**) and zeta-potential (**c**). Conjugates differed by the amount of bound PMX B: 1–280 μg/mg polymer; 2–312 μg/mg polymer; 3–402 μg/mg polymer (for other details see Table 2). All measurements were performed in 0.01 M sodium phosphate buffer (pH 7.4).

Given that P(Glu-*co*-Phe) and its conjugates with PMX B are self-assembled into nanoparticles, their hydrodynamic diameter (*D_H_*) and polydispersity index (*PDI*) were estimated by dynamic light scattering (DLS) and nanoparticle tracking analysis (NTA). The results obtained are shown in Figure 3. According to both DLS and NTA, the mean hydrodynamic diameter was slightly increased when PMX B was conjugated with P(Glu-*co*-Phe) nanoparticles. However, there was no statistically significant difference in *D_H_* when the amount of conjugated PMX B was increased from 280 to 400 μg/mg polymer. In general, the mean hydrodynamic diameter values determined by NTA were lower (up to 250 nm) than those measured by DLS (up to 350 nm). This may be a result of the higher sensitivity of NTA to smaller nanoparticles and a different way of averaging the data. All nanoparticles demonstrated quite narrow dispersity before and after conjugation (PDI ˂ 0.3). As expected, the nanoparticles had a negative surface zeta-potential (−28 mV), which slightly increased after PMX B conjugation (around −21 mV). This can be explained by the participation of part of the surface carboxylic groups in the conjugation, as well as partial compensation of the surface charge with positively charged PMX B. 

### 2.3. Synthesis and Characterization of Conjugates Based on Modified PVSI

One of the possible and easy ways to modify PVSI is alkaline hydrolysis of the N-succinimide ring accompanied by its opening with the liberation of a free carboxyl group (Figure 2b). Since PVSI is a hydrophobic polymer insoluble in water, the alkaline hydrolysis reaction was carried out in a mixture of aqueous sodium hydroxide solution with dioxane at 22 °C for 4 h. The selected conditions are mild and allow the hydrolysis of N-vinylsuccinimide (VSI) to vinyl succinamic acid (VSAA). The modification was testified by ^1^H NMR spectroscopy (DMSO-d_6_, 25 °C) by a decrease in intensity of the signal at 3.1–3.6 ppm corresponding to CH_2_ groups of VSI ring and appearance of the signals at 3.6–4.1 ppm belonging to CH_2_ groups of VSAA (Figure S3, Supplementary Materials). According to ^1^H NMR spectroscopy, the rate of hydrolysis was 70 mol%. Thus, the modified polymer represents a random copolymer of VSAA and VSI (P(VSAA-*co*-VSI)). Being amphiphilic, the obtained copolymer did not provide true solutions in water giving the dispersions of nanoparticles. 

Synthesis of PMX B conjugates with P(VSAA-*co*-VSI) was performed according to the same scheme as described above for polypeptides, since P(VSAA-*co*-VSI) carboxyl groups were used for conjugation (Figure 1). The conjugates obtained were analyzed by ^1^H NMR spectroscopy. Signals in the range of 0.25–1.5 ppm corresponding to PMX were detected in the spectra of polymer conjugates (Figure S4, Supplementary Materials). The amounts of PMX B used for conjugation and conjugation efficacy are provided in Table 3. The conjugation efficacy for P(VSAA-*co*-VSI) was decreased with the increase in the initial PMX B amount taken for conjugation. In general, the conjugation efficacy and its trend for P(VSAA-*co*-VSI) were the same as for other carboxylate polymers (PGlu and P(Glu-*co*-Phe)). The decrease in conjugation efficacy most probable is due to an increase in steric hindrances when the amount of conjugated PMX B increases. 

**Figure 4:**
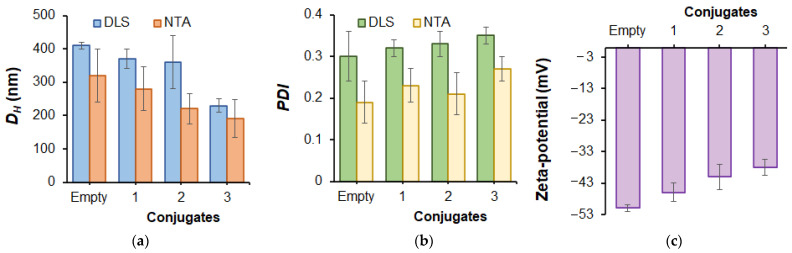
Characteristics of self-assembled empty P(VSAA-*co*-VSI) and PMX B-conjugated nanoparticles determined by dynamic light scattering, nanoparticle tracking analysis and electrophoretic light scattering: hydrodynamic diameter (*D_H_)* (**a**), polydispersity index (PDI) (**b**) and zeta-potential (**c**). Conjugates were differed by the amount of conjugated PMX B: 1–282 μg/mg polymer; 2–368 μg/mg polymer; 3–415 μg/mg polymer (for other details see Table 3).

Neat and conjugated polymer nanoparticles were characterized by DLS, NTA and electrophoretic light scattering (ELS) to determine *D_H_*, *PDI* and zeta-potential, respectively (Figure 4). In the case of P(VSAA-*co*-VSI), the formed neat nanoparticles were slightly larger than P(Glu-*co*-Phe), despite the close hydrophobic fragment content (25 and 30 mol%, respectively). Since both copolymers are negatively charged, the repulsion of the charged polymer chains takes place in both cases. Thus, the difference in hydrodynamic diameters of nanoparticles based on P(VSAA-*co*-VSI) and P(Glu-*co*-Phe) may be explained by two reasons. First, the higher hydrophobicity of Phe units compared to VSI leads to nanoparticle compaction due to hydrophobic interactions within the nanoparticle [32]. Furthermore, the aromatic rings of Phe can participate in π–π interactions. Second, it is known that polypeptides can provide ordered fragments by forming secondary structures (α-helices and β-sheets), which can also lead to a more compact packing of polymer chains inside the nanoparticle. At the same time, disordered PVSI coils form looser structures.

P(VSAA-*co*-VSI)-based nanoparticles demonstrated a slightly higher dispersity (PDI *˂* 0.4) than P(Glu-*co*-Phe) ones. Similar to the P(Glu-*co*-Phe)-based conjugates, zeta-potential increased after conjugation of PMX B with P(VSAA-*co*-VSI).

### 2.4. Synthesis and Characterization of Conjugates Based on Modified PMAG

PMAG is a nontoxic, non-charged and water-soluble polymer [33,34]. For covalent modification of this polymer with amino-bearing molecules (amino acids, peptides, dyes, etc.), the generation of reactive functionality is a required step. There are many ways to modify sugar hydroxyls, but one of the key methods is the oxidizing of vicinal diols with sodium periodate (Figure 2c). In this work, we used a previously developed protocol allowing the controllable oxidation of glucose hydroxyls in PMAG with the generation of 10 mol% of aldehyde groups [33,34]. The reaction was carried out in water at 4 °C for 24 h. Other details can be found in Section 3.2.3 of Materials and Methods.

Conjugation of PMX B with PMAG was carried out in water at 22 °C for 2 h. PMX B in formed conjugate was linked to the polymer via aldimine bond, known also as a Schiff base. In contrast to amide bond, the aldimine one is labile and can hydrolyze in aqueous media with a higher rate at slightly acidic pH [35,36]. Thus, such conjugates can be considered as systems for gradual release of free antibiotic. In this work, we have studied two variants of conjugates: those linked with aldimine bonds and a secondary amine group produced after reduction of a Schiff base with sodium borohydride (Figure 2c). 

The conjugates obtained were testified by ^1^H NMR spectroscopy. The signals corresponding to PMX B were found in the spectra of polymer conjugates (Figure S5, Supplementary Materials). The amount of PMX B used for conjugation and the effectiveness of conjugation for the reduced conjugates are shown in Table 4. As for the activated esters, conjugation through the aldehyde groups was also effective. The PMAG−PMX B conjugates were water-soluble.

### 2.5. Study of Polymyxin B Release from Conjugates 

To estimate the rate of PMX B release from the conjugates, a PMAG−PMX B conjugate, in which PMX B was bound to a polymer via the Schiff base, was used for the study. Since both P(Glu-*co*-Phe)−PMX B and P(VSAA-*co*-VSI)−PMX B conjugates have slowly hydrolysable amide bonds between the polymer and antibiotic, we selected P(Glu-*co*-Phe)−PMX B conjugate to compare the release rate of PMX B with the encapsulated system based on the same polymer and developed in our previous study [28]. Freshly prepared, rapidly purified, frozen, and lyophilized conjugates were dissolved/redispersed immediately prior to the release study. The solutions of PMAG–PMX B conjugate and dispersion of P(Glu-*co*-Phe)−PMX B in 0.01 M phosphate buffer with pH 7.4 and 5.8 were prepared and incubated at 37 °C during one week. While pH 7.2–7.4 is the normal physiological pH of the blood, acidification to pH 5.2–6.0 is common in areas of tissue damage and inflammation [37]. 

**Figure 5:**
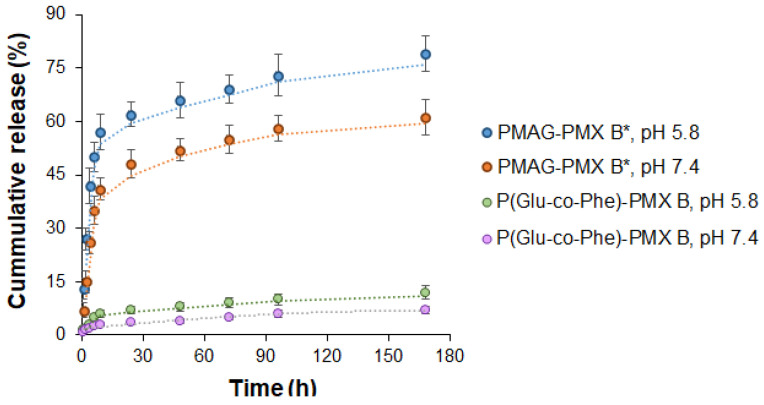
Release of PMX B from conjugates with PMAG (* in the form of Schiff base) and P(Glu-*co*-Phe) in 0.01 M sodium phosphate buffer with pH 7.4 or 5.8 at 37 °C.

As can be seen from Figure 5, the release at slightly acidic pH was faster than at pH 7.4. In the case of PMAG−PMX B, more than half (57%) of PMX B conjugated to PMAG was released after 9 h in the buffer with pH 5.8. In comparison, the same release at pH 7.4 (58%) was achieved after 4 days of incubation.

As expected, the release from the amide-bound PMX B conjugate was much slower. For the P(Glu-*co*-Phe)−PMX B conjugate, PMX B release equal to 7 and 12% was achieved during incubation in media with pH 7.4 and 5.8, respectively. This was much slower than for the recently developed PMX B encapsulated P(Glu-*co*-Phe)-based systems [28]. However, the obtained results were in agreement with the data published for another amide-linked PMX conjugate. To compare, recently, Dubashynskaya et al. reported that the release of colistin from the conjugate with amide-bonded hyaluronan was no more than 3% in buffer at pH 7.4 and 5% in buffer at pH 5.2 [21] within 24 h. Chiron et al. studied the release of colistin from a conjugate with succinylated dextrin [16]. Total release from the conjugate in buffer (pH 7.4) at 37 °C was observed within 3 months. Addition of amylase to the buffer medium triggered a more active release of colistin. Depending on the degree of substitution (1.1–8.3 mol%), 40–80% release of colistin induced by enzymatic hydrolysis was observed within 48 h of incubation at 37 °C [18]. In all cases, the conjugates revealed antimicrobial activity.

### 2.6. Conjugation of Deferoxamine and Synthesis of Complex Conjugates

In order to compare the conjugation efficacy of DFOA to PMX, the conjugation of DFOA to P(Glu-*co*-Phe) was studied under variation of the initial amount of DFOA relative to the polymer and keeping the conjugation conditions as for PMX B. The results on DFOA conjugation are presented in Table 5. As seen, the DFOA conjugation efficacy was approximately two times lower than that for PMX B. Most likely, this result is explained by statistical probability, since PMX B contains five reactive amino groups, while DFOA contains only one. At the same time, DFOA is less sterically hindered than PMX B.

Based on the results of testing the antimicrobial activity of the PMX B polymer conjugates (Section 2.6), two complex conjugates of PGlu and PMAG with both PMX B and DFOA were prepared and evaluated against *P. aeruginosa*. The characteristics of both conjugates are shown in Table 6. Both components were effectively conjugated to both polymers with the expected higher efficacy for PMX B. 

### 2.7. Biological Evaluation

#### 2.7.1. Cytotoxicity of Polymers, Polymyxin B and Their Conjugates

**Figure 6:**
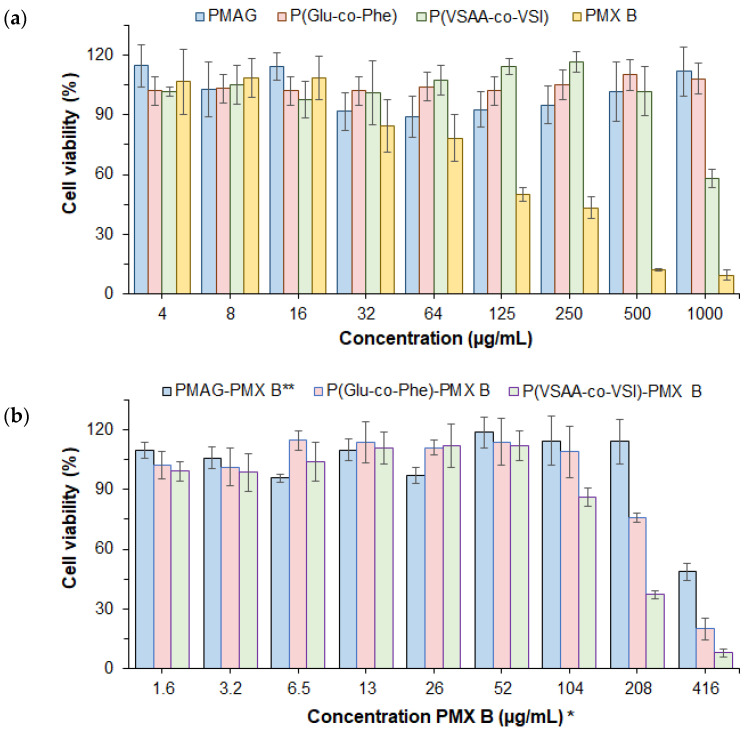
Viability of HEK 293T cells incubated in the presence of free polymers and polymyxin B (**a**) and their conjugates (**b**) for 48 h (* conjugates were tested relative to PMX B concentration; ** PMAG−PMX B conjugate was tested with the reduced aldimine bonds). Data are presented as mean ± SD (n = 4).

Taking into account the known nephrotoxicity of PMX B, the cytotoxicity of the polymers, free PMX B and their conjugates was examined in human embryonic kidney cells (HEK 293T cell line). The cell viability was determined after 48 h using the MTT assay (Figure 6). All polymers used as carriers for conjugation demonstrated high biocompatibility with cells (Figure 6a, Table 7). In particular, half-minimal inhibitory concentrations (IC_50_) were higher than 1000 μg/mL for PMAG and P(Glu-*co*-Phe), and 500 μg/mL for P(VSAA-*co*-VSI). At the same time, free PMX B exhibited a more pronounced cytotoxicity (Figure 6a, Table 7). 

Since the cytotoxicity of free PMX B was much higher than that of the polymers, and to compare the IC_50_ for free and conjugated PMX B, the polymer conjugates were tested relative to the PMX B concentration. The conjugation of PMX B with polymers indeed reduced the PMX B cytotoxicity (Figure 6b, Table 7). Increased cell viability compared to free PMX B was revealed for all types of conjugates. The same trend was previously reported by Dubashinskaya et al. for the polymyxin E conjugates with hyaluronic acid [21]. 

A comparison of the different conjugates led to the conclusion that the PMAG- and P(Glu-*co*-Phe)-based conjugates were the least toxic (Table 7). These polymers also showed the highest biocompatibility with the cells.

#### 2.7.2. Comparison of Antimicrobial Activity of Conjugates

**Figure 7:**
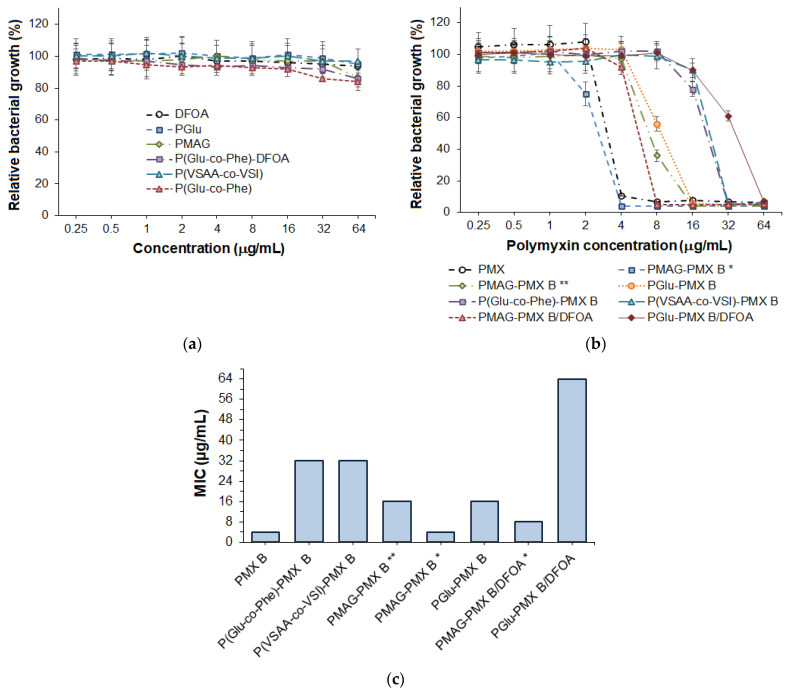
Antimicrobial activity of polymers and DFOA (**a**), and free and conjugated PMX (**b**) depending on concentration as well as the comparison of minimal inhibition concentration for different antibacterial samples (**c**) (* aldimine bonds (Schiff base) between polymer and PMX; ** reduced aldimine bonds). *P. aeruginosa* was cultured in 96-well plates with and without testing samples for 18 h, and then the optical density (OD) proportional to bacterial growth was measured at 630 nm. Relative bacterial growth (%) was determined as a ratio of the OD_630_ in each concentration of the testing samples to OD_630_ in the control (0 μg/mL). Data are presented as the mean ± SD (n = 9).

The antimicrobial activity of the free polymers and their conjugates with PMX B were evaluated against *P. aeruginosa*. From the results presented in Figure 7a, one can see that neither polymers nor DFOA exhibited antimicrobial activity in the concentration range of 0.25–64 μg/mL. At the same time, as seen from Figure 7b, PMX B conjugates expectedly showed antimicrobial activity (Figure 7b). A comparison of the MICs determined for free PMX B and its conjugates is shown in Figure 7c. 

The best antimicrobial activity (4 μg/mL), equal to the MIC of free PMX B, was observed for the PMAG–PMX B conjugate, where the antibiotic was bound to the polymer via an aldimine bond (Schiff base). Reduction of the aldimine bond contributed to a fourfold increase in the MIC (to 16 μg/mL). This fact can be explained by the faster release of PMX B from PMAG-based conjugates with hydrolysable bonds. Reduced microbial properties (MIC = 32 μg/mL) were found for P(Glu-*co*-Phe)−PMX B and P(VSAA-*co*-VSI)−PMX B conjugates present as nanoparticles. In turn, the PGlu-based conjugate, as well as the reduced PMAG−PMX conjugate, showed twice as low MIC as the nanoparticle-based conjugates. The most probable reason is that PMX B conjugated with amphiphilic polymers hides inside the nanoparticles, due to hydrophobic interactions of the phenylalanine residue and the aliphatic tail of PMX B with the hydrophobic part of the nanoparticle. Recently, such a property has been demonstrated for the non-covalent complexes of P(Glu-*co*-Phe) and PMX B [28].

Thus, the best antimicrobial properties were observed for the water-soluble PMX B conjugate with a hydrolysable bond. Intermediate properties were shown by PMAG- and PGlu-based PMX B conjugates with stable (secondary amine) or slowly hydrolysable (amide) bonds, respectively. The worst antimicrobial activity was found for PMX B conjugates based on amphiphilic copolymers with slowly hydrolysable amide bonds. 

According to the published works, many factors affect the MIC for conjugates linked with amide bonds. For example, Dubashynskaya et al. observed the dependence of MIC on the degree of substitution (DS). In particular, when the DS was 8%, the MIC against *P. aeruginosa* for hyaluronan–colistin conjugates was 1 µg/mL, the same as for free colistin. At the same time, a decrease in DS to 5 and 3% was accompanied with the MIC increase to 4 and more than 8 µg/mL, respectively [21]. Stokniene et al. demonstrated that depending on the conjugation conditions, the MIC for various amide-linked PMX B and E conjugates with oligosaccharides was the same as that of the free antibiotic or two to four times higher [17]. An increase in the MIC for P(PEG-acrylate)-colistin conjugates against *Acinetobacter baumannii* (Ab ATCC 19606) to 8–32 µg/mL compared to 1 µg/mL for free colistin was observed by Zhu et al. [20].

A study of PMX-polymer conjugates containing DFOA revealed a decrease in antimicrobial properties. For PMAG–PMX B/DFOA, an increase in MIC from 4 to 8 μg/mL was observed compared to the PMAG–PMX B conjugate. In turn, for PGlu-PMX B/DFOA, an increase in MIC from 16 to 64 μg/mL was detected. Overall, the MIC of PMX B of 8 μg/mL is an appropriate result. Regarding conjugates, the MIC for PMX conjugates with siderophores depends on the bacterial strain, the antibiotic type, the linkers in the conjugates, and the presence and absence of Fe^3+^ ions in the medium. For example, Boyce et al. reported that no improvement in the antimicrobial properties was observed for siderophore conjugates with the peptide antibiotic daptomycin linked through L- and D-derivatives of hexapeptide against *Staphylococcus aureus.* However, some of the conjugates demonstrated the improvement in antimicrobial activity for *Escherichia coli* [38]. Suoto et al. studied the antimicrobial properties of norfloxacin and its conjugates with siderophore. Despite the four tested conjugates proving clear antibacterial activity, their activity was lower than for free norfloxacin. The best result for conjugate was a 16-fold worth compared to free antibiotic. The authors speculated that the terminal amine of siderophore (vanchrobactin analogue) used for conjugation might be critical for the recognition properties [39].

Wencewicz et al. showed that the same conjugates with siderophores can demonstrate a different behavior in the absence and in the presence of Fe^3+^ ions [40]. A series of conjugates of loracarbef (cephalosporins) and ciprofloxacin (fluroquinolones) with sideromycins (siderophores) was synthesized and tested against a panel of ESKAPEE bacteria (*Enterococcus faecium, S. aureus, Klebsiella pneumoniae, A. baumannii, P. aeruginosa, Enterobacter aerogenes, E. coli*). The conjugates of both antibiotics with siderophore revealed a reduced spectrum of activity compared to the broadly active parent antibiotics. The strongest activity for both conjugated and free antibiotics was found against *S. aureus*. However, depending on the conjugate composition, the MICs for the loracarbef–siderophore conjugates were 32–128 µM versus 1 µM for free antibiotic, while for the ciprofloxacin–siderophore conjugates, these values were 32–64 µM in comparison to 0.5 µM for free antibiotic. At the same time, under Fe^3+^ conditions, a fourfold improvement in MIC was detected for ciprofloxacin–siderophore conjugates [40]. 

In our case, PMX B conjugates based on PMAG with and without DFOA obviously seem to be the most promising. However, further in-depth study is necessary to optimize the content of conjugated PMX B and DFOA, as well as testing against other Gram-negative bacteria in the absence and in the presence of Fe^3+^ ions.

## 3. Materials and Methods

### 3.1. Materials 

Polymyxin B, N-hydroxysuccinimide (NHS, 98%), 1-ethyl-3-(3-dimethylaminopropyl)carbodiimide (EDC, ≥98%) and sodium periodate (extra pure) were purchased from Sigma-Aldrich (Darmstadt, Germany). Deferoxamine was a product of Novartis Pharma (Basel, Switzerland). All components used for the preparation of buffer solutions were purchased from Vecton (St. Petersburg, Russia) and were of analytical grade of purity. 

For additional purification of buffer solutions, they were filtered through Millipore Merck membrane filters (Darmstadt, Germany) with a pore diameter of 0.22 μm. Solutions for chromatographic analysis were filtered through syringe-microfilters of 0.45 μm and 1 μm (PVDF, FilterBio, Nantong, China). Dialysis cellulose membranes (MWCO: 1000, 2000; Orange Scientific, Braine-l’Alleud, Belgium) were used for polymer and conjugate purification. For ultrafiltration, Amicon Ultra 0.5 and Eppendorf concentration tubes (MWCO: 3000, 5000; Sigma-Aldrich, Darmstadt, Germany) were used.

*P. aeruginosa* (ATCC 27853) from Museum of Microbiological Cultures of State Research Institute of Highly Pure Biopreparations FMBA (St. Petersburg, Russia) and Müller–Hinton Broth (HiMedia, Mumbai, India) were used to study the antibacterial effect of PMX B and its conjugates. HEK 293T cells were purchased from the cell culture collection of the Institute of Cytology of Russian Academy of Sciences (St. Petersburg, Russia). 

### 3.2. Methods

#### 3.2.1. Polymer Synthesis and Characterization 

Poly(L-glutamic acid-*co*-L-phenylalanine) and Poly(L-glutamic acid) were synthesized by ring-opening polymerization of N-carboxyanhydrides of α-amino acids as described earlier [32]. Poly(2-deoxy-2-methacrylamido-D-glucose) and poly(N-vinylsuccinimide) were synthesized by RAFT polymerization according to protocols published elsewhere [30,31]. For PGlu, P(Glu-*co*-Phe) and PMAG, degree of polymerization was determined by ^1^H NMR spectroscopy. Calculation of the degree of polymerization was performed after the polymer was purified from low molecular weight impurities. For PGlu, DP was calculated by the ratio of the integral signal intensities of methylene protons of the benzyl group (δ = 5.0–5.2 ppm) in precursor poly(glutamic acid γ-benzyl ester) and methyl protons of hexylamine used as initiator (δ = 0.84 ppm). For P(Glu-*co*-Phe), DP was calculated as a ratio of the integral signal intensities of methylene protons of the benzyl group (δ = 5.0–5.2 ppm) in protected polymer (Glu units) and phenylalanine aromatic protons signals (δ = 6.7–7.4 ppm) (Phe units) to methyl protons of hexylamine used as initiator (δ = 0.84 ppm). In the case of PMAG, the signals of the aromatic ring of the RAFT-agent (δ = 7.4–7.7) ppm and the total proton signals of the glucose ring (δ = 3.3–4.2 ppm) were used as reference signals. Other details are given as footnotes to Table 1.

#### 3.2.2. Synthesis of PGlu- and P(Glu-*co*-Phe)-Based Conjugates of Polymyxin B 

A sample of the lyophilized (co)polymer was dissolved/dispersed in distilled water using an ultrasonic probe (30 s) at concertation of 1 mg/mL and then placed on an ice bath under stirring. To the cooled suspension, an aliquot of N-hydroxysuccinimide (NHS) solution in water was added at 2*n* (mol), where *n* (mol) was calculated for 10 mol% of glutamic acid units in polymer. The reaction was left for 20 min, after which an aliquot of 1.2*n* (mol) water-soluble carbodiimide (EDC) solution was added and the reaction mixture was left for 40 min. Next, the suspension was removed from the ice bath and a freshly prepared solution of PMX (0.3–0.7*n*) in borate buffer solution with pH 9.2 was dropped into the solution of activated polymer under stirring and then left for 2 h at 22 °C. After that, the solution/dispersion was transferred to an ultrafiltration concentrator tube and centrifuged with 0.1 M phosphate–saline buffer (pH 7.0) several times to remove the unbound PMX. Several filtrate portions with unbound PMX were combined together and lyophilized for further HPLC analysis (see Section 3.2.5). The purified dispersion was placed in a dialysis tube with MWCO 3000 and additionally purified against 0.01 M PBS, pH 7.0, and water for 24 h. Afterwards, the conjugates were freeze-dried and stored at 4 °C. Characterization of conjugates by ^1^H NMR spectroscopy was carried out in DMSO-d_6_ at 25 °C. To prepare the dispersion of nanoparticles, a weighted portion of polymer sample was dispersed in distilled water or sodium phosphate buffer and ultrasonicated with the use of Sonopuls MS 73 homogenizer at 20% power for 15 to 30 s. After that, the dispersion was left for 20–30 min at room temperature and then analyzed with use of ZetaSizer Nano ZS (*D_H_*, *PDI*, zeta-potential) and NanoSight NS300 (*D_H_*, *PDI*) analyzers (Malvern, UK). The measurements were carried out under thermostatic conditions (25 °C) in three parallel runs for each sample.

Conjugation efficacy was calculated using the following equation:

(1) $$Conjugation\;efficacy=\frac{m_{conj.}}{m_{0}}\;100\%$$

where *m_conj._* is a mass of conjugated antibiotic calculated as a difference between *m*_0_ and the mass of unbound antibiotic, and *m*_0_ is an initial mass of antibiotic taken for the reaction. 

#### 3.2.3. Synthesis of PMAG-Based Conjugates of Polymyxin B

For conjugation, it was necessary to generate the reactive functionality in PMAG. This was performed by partial oxidation of glucose units with sodium metaperiodate according to the previously developed procedure [34]. Briefly, PMAG was dissolved in distilled water and cooled to 4 °C. While stirring, NaIO_4_ was added in the molar ratio [MAG]/[NaIO4] = 0.3 to form 10 mol% aldehyde groups. The reaction was carried out in the dark for 24 h at 4 °C. After that, the product was purified by ultrafiltration with distilled water. 

An aliquot of freshly prepared PMX B solution in borate buffer at pH 9.2 was added by drops to the partially oxidized PMAG sample; the reaction was left for 2 h under stirring at 22 °C. To reduce aldimine bonds, a NaBH_4_ aqueous solution with a concentration of 2 mg/mL was added and left for 1 h. After that, purification was carried out using ultrafiltration with distilled water in MWCO 3000 concentrator tubes. Several filtrate portions with unbound PMX were mixed together and lyophilized for further HPLC analysis (see Section 3.2.5). The conjugate was freeze-dried and stored at 4 °C. Characterization of conjugates by ^1^H NMR spectroscopy was carried out in DMSO-d_6_/D_2_O (50/50, *v/v*) at 25 °C. 

#### 3.2.4. Synthesis of P(VSAA-*co*-VSI)-Based Conjugates of Polymyxin B

P(VSAA-*co*-VSI) was prepared by partial hydrolysis of PVSI. Briefly, a sample of polymer was dissolved in 1,4-dioxane under intensive stirring; 2.5% NaOH solution was added at 22 °C and left for 4 h. After the reaction, the basic medium was neutralized with 0.1 M HCl. The copolymer was purified by dialysis against DMF/water and finally, deionized water. The purified P(VSAA-*co*-VSI) was freeze-dried. The generated carboxyl groups were activated using NHS and EDC and conjugated with PMX as described in Section 3.2.1. Other procedures such as purification, characterization and storage were the same as for P(Glu-*co*-Phe) (Section 3.2.2). 

#### 3.2.5. Synthesis of Mixed Conjugates 

Mixed conjugates containing both PMX and DFOA were obtained using the same methodology as described in Section 3.2.2 and Section 3.2.3 for copolymers bearing carboxylic and aldehyde groups. First, the activation of the carboxyl group of L-Glu units was performed, then aliquots of solutions of PMX and DFOA at 22 °C were simultaneously added at the required content. After that, purification was carried out using ultrafiltration with 0.1 M PBS (pH 7.0) in MWCO 3000 concentrator tubes. Several filtrate portions with unbound PMX B were mixed together and lyophilized for further HPLC analysis (see Section 3.2.6). The conjugate was freeze-dried and stored at 4 °C.

#### 3.2.6. HPLC Analysis of Polymyxin and Deferoxamine

Quantitative analysis of the PMX and DFOA was performed using ion-exchange HPLC using a Prominence LC-20AD system (Shimadzu, Kyoto, Japan) equipped with a diode matrix detector and an ultra-short monolithic analytical CIM-SO_3_ column (5 × 5 mm) (BIA Separations, Ljubljana, Slovenia). Buffer solutions were used as the mobile phases: A—0.005 M sodium phosphate buffer solution, pH 7.0; B—2 M NaCl. Analysis was performed under binary gradient conditions: 0–2 min—100% A, 2–7 min—0 to 100% B, 7–12 min—100% B. The mobile phase flow rate was 0.5 mL/min. Detection was performed at 215 nm. DFOA and PMX B retention times were 3.5 and 7.5–7.9 min, respectively. The quantification was carried out regarding a calibration plot pre-built for PMX and DFOA in the range of concentration 0.05–5.00 mg/mL and 0.05 to 2.00 mg/mL, respectively. 

#### 3.2.7. In Vitro Release Study

An amount of 1 mg of conjugate was dissolved/redispersed in a 1 mL of 0.01 M phosphate buffer solution with pH 7.4 or 5.8 and incubated at 37 °C. At predetermined intervals, the buffer medium was replaced by ultracentrifugation using a 3000 MWCO Vivaspin concentrators with the fresh buffer portions. The concentrations of the released PMX B in the supernatant was determined by quantitative HPLC using the preliminary built calibration curve (see Section 3.2.6). The release in % was calculated as a ratio of the released cumulative mass of PMX B to mass of conjugated PMX B. The conjugates with similar loading were used for the release study: 316 ± 21 µg PMX B/mg polymer for PMAG−PMX B and 312 ± 19 µg PMX B/mg polymer for P(Glu-*co*-Phe)−PMX B. 

#### 3.2.8. In Vitro Cytotoxicity Study

HEK 293T cells were cultivated in DMEM/F12 medium with 10% FCS, 2 mM glutamine, and 1% gentamicin (Sigma-Aldrich, Darmstadt, Germany). In 96-well plates, 3.5 × 10^3^ cells in 100 µL were seeded per well and left for adhesion at 37 °C for 24 in a humidified 5% CO_2_ atmosphere. After that, the medium in the wells was replaced with 200 µL of solution containing the test samples at the specified concentrations (*n* = 4) and left for incubation under the same conditions for 48 h. Then, the medium was removed and 100 µL of MTT reagent solution (5 mg/mL stock solution) was added to each well. The plates were incubated in the CO_2_ incubator for 3 h at 37 °C. Finally, the solution in the well was replaced with 50 µL of DMSO to dissolve formazan crystals and the optical density in each well was measured at 570 nm using fluorescent plate reader (Fluoroscan Ascent, Thermo Fisher Scientific Inc., Waltham, MA, USA). The wavelength of 690 nm was used to measure the background absorbance. The data obtained were normalized as a percentage of the control. Free polymers and PMX B were tested at concentrations ranging from 4 to 1000 µg/mL. The PMX B polymer conjugates were tested at concentrations in the range of 1.6–416 µg/mL regarding PMX B. Non-linear curve fitting/growth/sigmoidal/dose–response functions (OriginPro 8.6) were used to calculate IC_50_ values from the concentration-dependent normalized cell viability data.

#### 3.2.9. Study of Antibacterial Activity

The minimum inhibitory concentration (MIC) of polymyxin B in the conjugates was determined by the microdilution method in Müller–Hinton broth. Before the samples were tested against *P. Aeruginosa*, they were checked for microbiological purity to exclude possible contamination. In all cases, the absence of microbial growth on Luria–Bertani agar was detected. To determine MIC of the samples, an overnight (18 h) culture of *P. aeruginosa* was used in the experiment. Inoculum in Müller–Hinton broth (125 μL) was added to the wells of a 96-well plate to reach the seeding density of 1 × 10^7^ CFU/mL. Müller–Hinton broth was only used as negative control (blank), *P. aeruginosa* culture as positive control (100% growth). Working solutions of the samples were prepared by diluting a stock solution in Müller–Hinton broth to prepare concentrations from 64 to 0.25 μg/mL. The plate was incubated for 18 h at 37 °C and then the optical density in the wells was measured at 630 nm using a microplate reader (ELx808™ Absorbance Microplate Reader, BioTek-Agilent, Santa Clara, CA, USA). Relative bacterial growth (%) was calculated from the data of OD measured for samples and control according to the equation:

(2) $$Relative\;bactearial\;growth=\frac{OD_{630}\;sample\;at\;Cx-OD_{630}\;blank}{OD_{630}\;positive\;control-OD_{630}\;blank}\times 100\%$$

where *Cx* is a certain concentration of the sample.

Each sample was tested three times in three independent series (n = 9). The plots in Figure 7 show the mean values ± SD for each type of samples.

## 4. Conclusions

All polymers used in this study were capable of effectively conjugating polymyxin B. The amphiphilic copolymers formed nanoparticles, while the homopolymers were water-soluble. All used polymer carriers were nontoxic to the human embryonic kidney cells (IC_50_ > 500 µg/mL), while free polymyxin B demonstrated a considerable cytotoxicity (IC_50_ = 130 µg/mL). In turn, PMX B conjugation with polymers contributed to a decrease in cytotoxicity. Depending on the polymer conjugate, the IC_50_ was in the range of 170–380 μg/mL. The best cell-compatibility was found for the PMAG-based conjugate with polymyxin B. 

Conjugates of polymyxin B with polymer nanoparticles were found to show reduced antibacterial activity compared to water-soluble conjugates and the free antibiotic. Among the two water-soluble polymers, the PMX B conjugate with neutral glycopolymer showed better antibacterial efficacy (MIC = 4 µg/mL) than the conjugate based on the negatively charged polymer (MIC = 16 µg/mL). Moreover, the MIC for the PMAG–PMX B conjugate matched the MIC determined for free PMX B. In the case of conjugates containing both PMX B and siderophore, PMAG-based systems also showed better results. Finally, the possibility of preparing PMAG-based conjugates with a hydrolysable or stable bond makes it possible to regulate the properties of the delivery systems. 

Summarizing the results of the biological evaluation of PMX B conjugates, it can be concluded that PMAG−PMX B conjugates with and without DFOA are the most promising among the other tested polymers. Compared to free PMX B, this type of conjugate shows reduced PMX B cytotoxicity combined with high antibacterial efficacy.

## Figures and Tables

**Table 1:** Structures and characteristics of polymers used as carriers for PMX B.

Structure	Name	*M_n_* ^a^	*Ð* ^d^	*DP* ^g^	Composition (mol%) ^b^	
					M_1_^i^	M_2_^i^
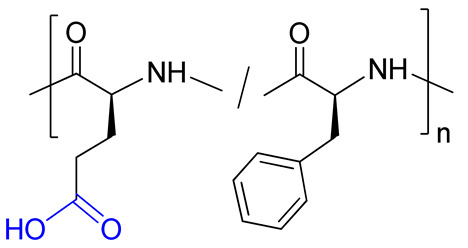	P(Glu-*co*-Phe)	6655 ^**b**^	1.20 ^**e**^	49 ^**b**^	75	25
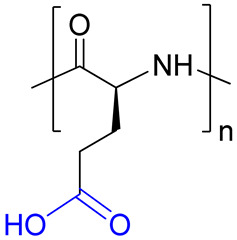	PGlu	6065 ^**b**^	1.40 ^**e**^	44 ^**b**^	−	−
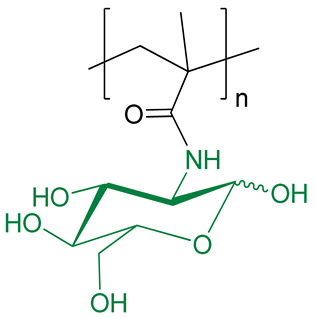	PMAG	20,040 ^**b**^	1.12 ^**f**^	80 ^**b**^	−	−
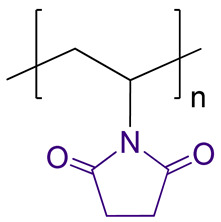	PVSI	14,300 ^**c**^	1.37 ^**f**^	112 ^**h**^	−	−

**^a^** *M_n_* is a number-average molecular weight; **^b^** Determined by ^1^H NMR spectroscopy (DMSO-d_6_, 25 °C for polypeptides and D_2_O, 60 °C for PMAG); **^c^** Determined by SEC with triple detection in tetrahydrofuran (THF) at 40 °C; **^d^** Ð is a polymer dispersity; **^e^** Determined for precursor γ-benzyl-protected polymers by SEC in *N,N*-dimethylformamide (DMF) containing 0.01 mol/L LiBr at 40 °C with refractometric detection using poly(methyl methacrylate) standards; **^f^** Determined by SEC with triple detection in DMF at 50 °C; ^**g**^ *DP* is a degree of polymerization; **^h^** Calculated from *M_n_* determined by SEC **^c^**; **^i^** M_1_ and M_2_ are the number of the units of monomer 1 and monomer 2, respectively, in the polymer chain.

**Table 2:** Efficacy of PMX B conjugation with PGlu or P(Glu-*co*-Phe) depending on the initial amounts of antibiotic.

Initial Amount of PMX B (µg/mg Polymer)	Amount of Conjugated PMX B (µg/mg Polymer)	Conjugation Efficacy (%)
*PGlu*		
200	194 ± 6	97 ± 3
300	288 ± 12	96 ± 4
400	335 ± 18	84 ± 6
500	430 ± 20	86 ± 4
*P(Glu-*co*-Phe)*		
300	280 ± 15	93 ± 5
400	312 ± 19	78 ± 5
500	382 ± 22	76 ± 4

**Table 3:** Efficacy of PMX B conjugation with P(VSAA-*co*-VSI) depending on the initial amounts of antibiotic.

Initial Amount of PMX B (µg/mg Polymer)	Amount of Conjugated PMX B (µg/mg Polymer)	Conjugation Efficacy (%)
300	282 ± 18	94 ± 6
400	368 ± 17	92 ± 4
500	435 ± 25	87 ± 5

**Table 4:** Efficacy of PMX B conjugation with ox-PMAG depending on the initial amounts of antibiotic (the results are presented for conjugates with reduced aldimine bonds).

Initial Amount of PMX B (µg/mg Polymer)	Amount of Conjugated PMX B (µg/mg Polymer)	Conjugation Efficacy (%)
300	290 ± 10	97 ± 3
400	316 ± 21	79 ± 5
500	425 ± 18	85 ± 4

**Table 5:** Efficacy of DFOA conjugation with P(Glu-*co*-Phe) depending on the initial amounts of siderophore.

Initial Amount of DFOA (µg/mg Polymer)	Amount of Conjugated DFOA (µg/mg Polymer)	Conjugation Efficacy (%)
200	96 ± 17	48 ± 8
300	105 ± 15	35 ± 5
500	210 ± 22	42 ± 4

**Table 6:** Efficacy of DFOA conjugation with different polymers depending on the initial amounts of siderophore.

Polymer	Amount of Conjugated Component (µg/mg Polymer)		Component Conjugation Efficacy (%)	
	PMX B	DFOA	PMX B	DFOA
PMAG	185	78	74	52
PGlu	198	70	79	48

**Table 7:** IC_50_ values for free polymers and PMX B, as well as their conjugates (HEK 293T cells, MTT, 48 h): * conjugates were tested relative to PMX B concentration; ** PMAG−PMX B conjugate was tested with the reduced aldimine bonds. Data are presented as mean ± SD.

Compound *	IC_50_ (µg/mL)
PMAG	>1000
P(Glu-*co*-Phe)	>1000
P(VSAA-*co*-VSI)	515 ± 4
PMX B	130 ± 14
PMAG–PMX B **	380 ± 5
P(Glu-*co*-Phe)-PMX B	220 ± 18
P(VSAA-*co*-VSI)-PMX B	170 ± 13

## Data Availability

Data available within the article or its supplementary materials.

## Supplementary Materials

The following supporting information can be downloaded at: https://www.mdpi.com/article/10.3390/ijms24031832/s1.
