# Supplementary material for: Polymyxin B Conjugates with Bio-Inspired Synthetic Polymers of Different Nature
Source: Int J Mol Sci. 2023 Jan 17;24(3):1832. doi: 10.3390/ijms24031832 (PMC9915011; doi:10.3390/ijms24031832)
Supplement: Supplementary file 1 [file ijms-24-01832-s001.zip › ijms-2075170-supplementary.pdf]

International Journal of
Molecular Sciences

MDPI

Supplementary Materials

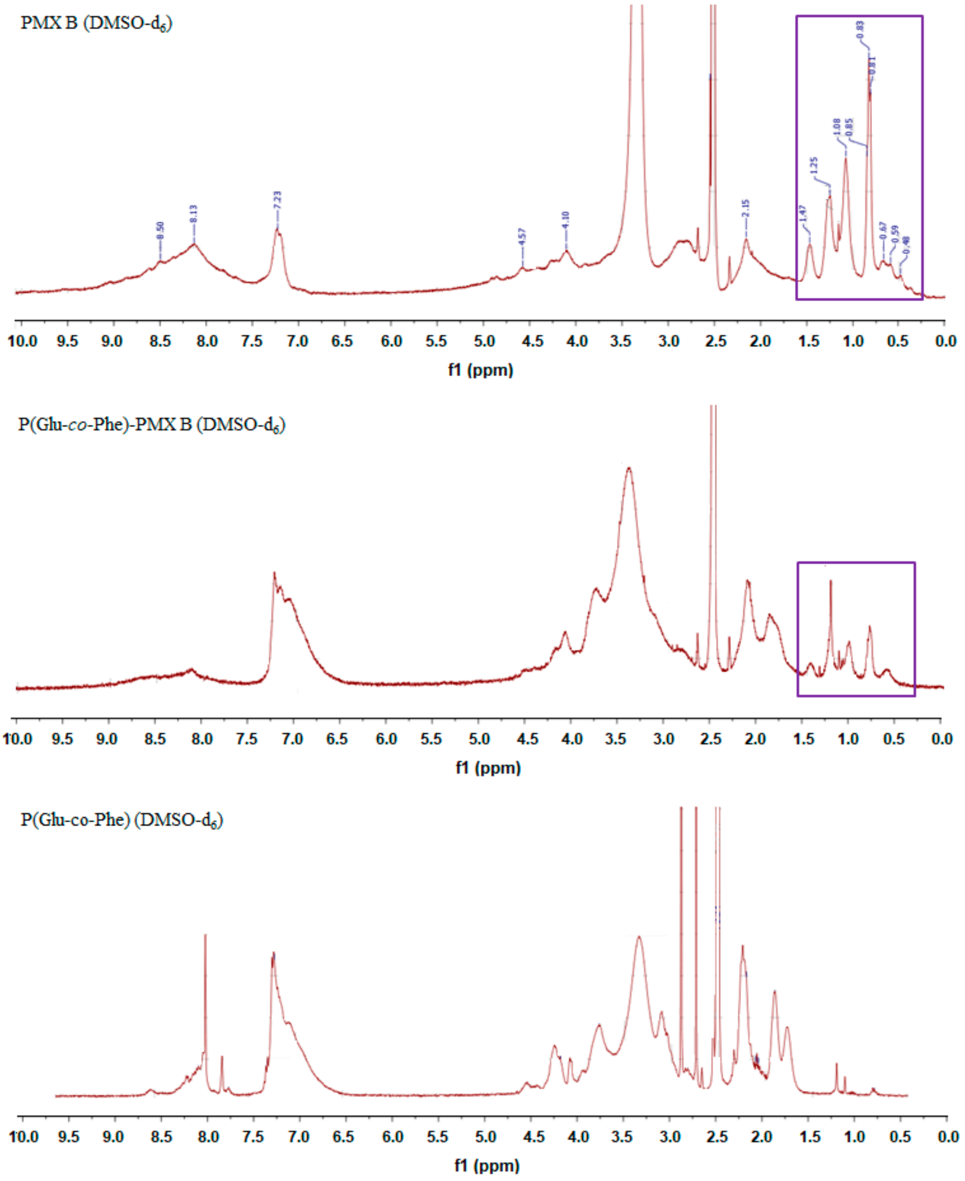

Figure S1. ^{1}H NMR spectra of free PMX B, P(Glu-*co*-Phe)-PMX B conjugate and P(Glu-*co*-Phe) (DMSO-d6, 25 °C). In the box, the signals of protons of $-CH_2$ and $-CH_3$ groups of PMX B cycle and aliphatic tail are marked.

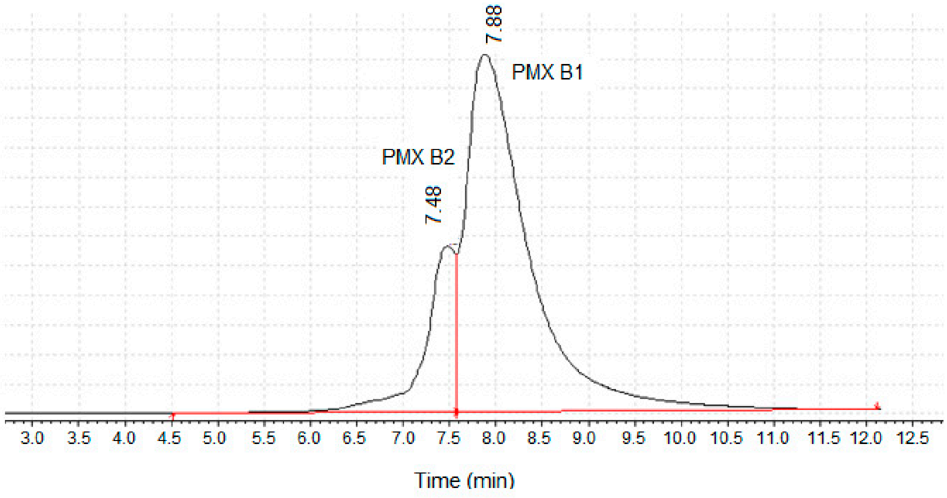

(a)

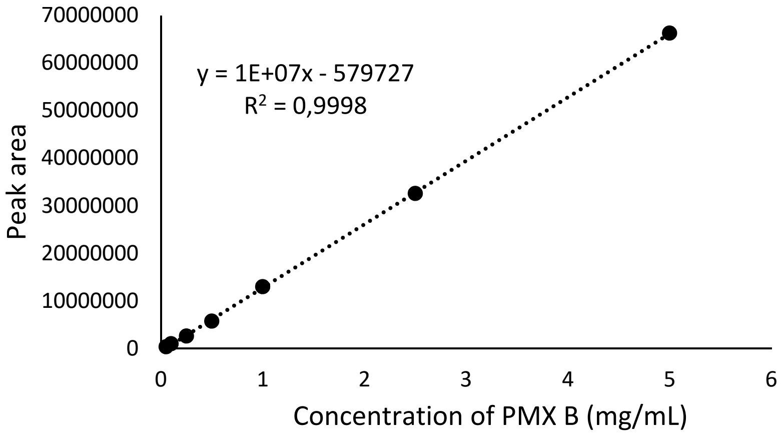

(b)

Figure S2. Chromatogram of PMX B **(a)** and calibration plot **(b)** obtained by ion-exchange HPLC (SO_3 monolithic disc).

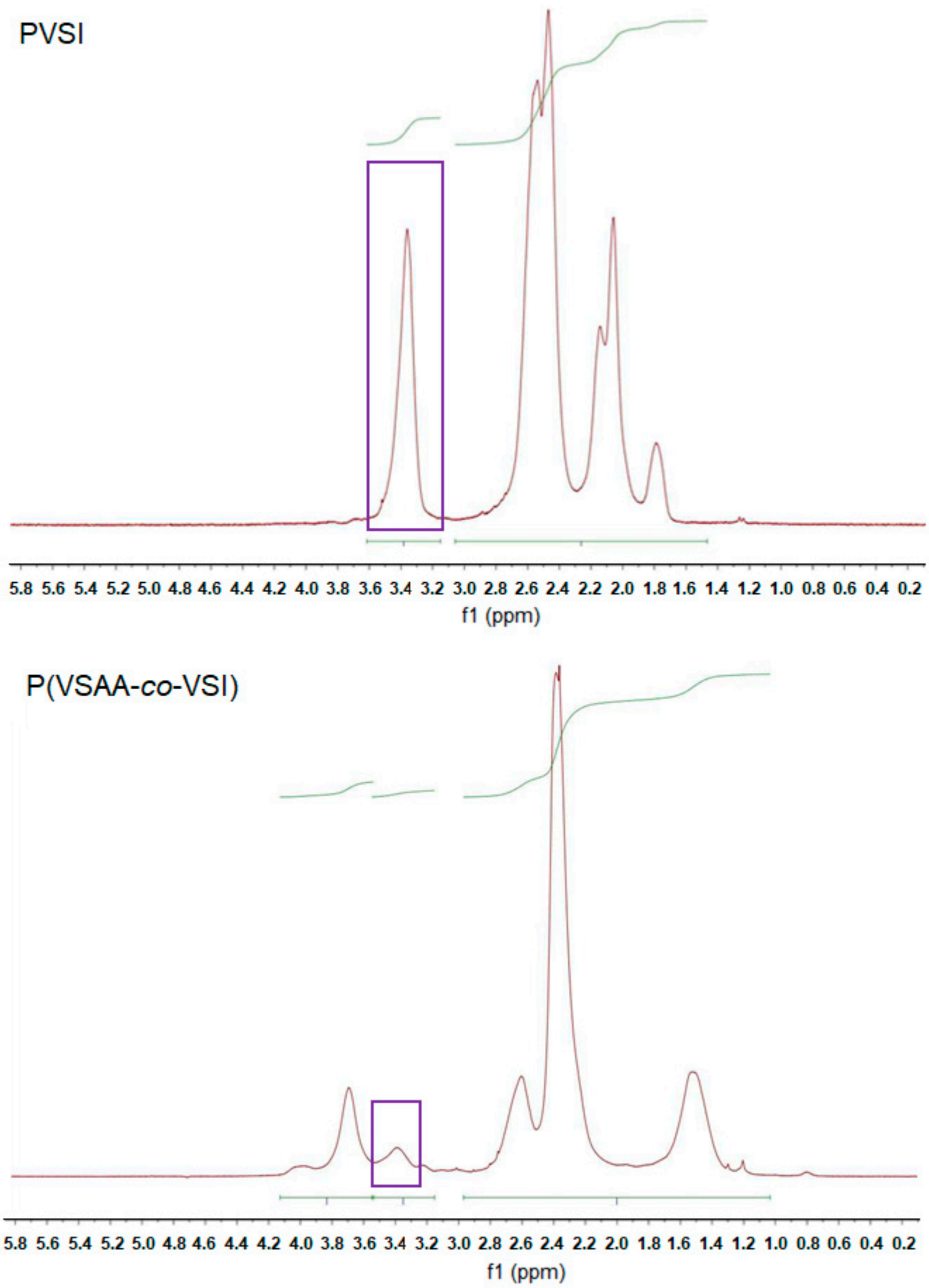

Figure S3. ^{1}H NMR spectra of PVSI and P(VSAA-*co*-VSI) obtained after PVSI alkaline hydrolysis (DMSO-d6, 25 °C). In the box, the signals of protons of -CH_2 groups in VSI cycle are marked. A significant decrease in the intensity of this signal indicates partial hydrolysis of the VSI ring.

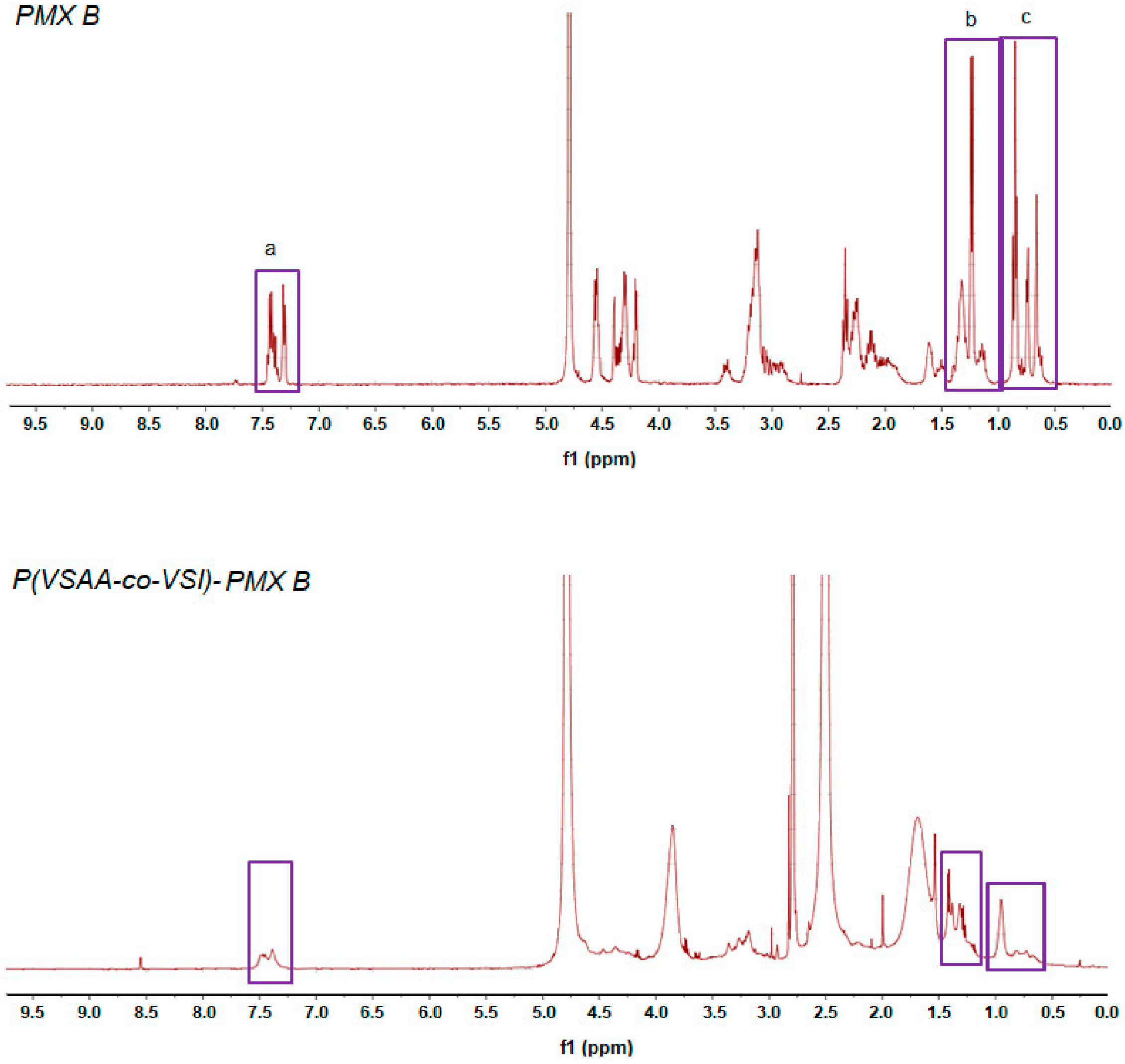

Figure S4. ^{1}H NMR spectra of P(VSAA-*co*-VSI)-PMX B conjugate (DMSO-d6/D_2O, 25 °C) and PMX B (D_2O, 25 °C). Signals in the boxes: *a* – aromatic protons from phenylalanine in PMX B; *b* and c – protons of terminal of -CH_2 and -CH_3 groups of PMX B cycle and aliphatic tail.

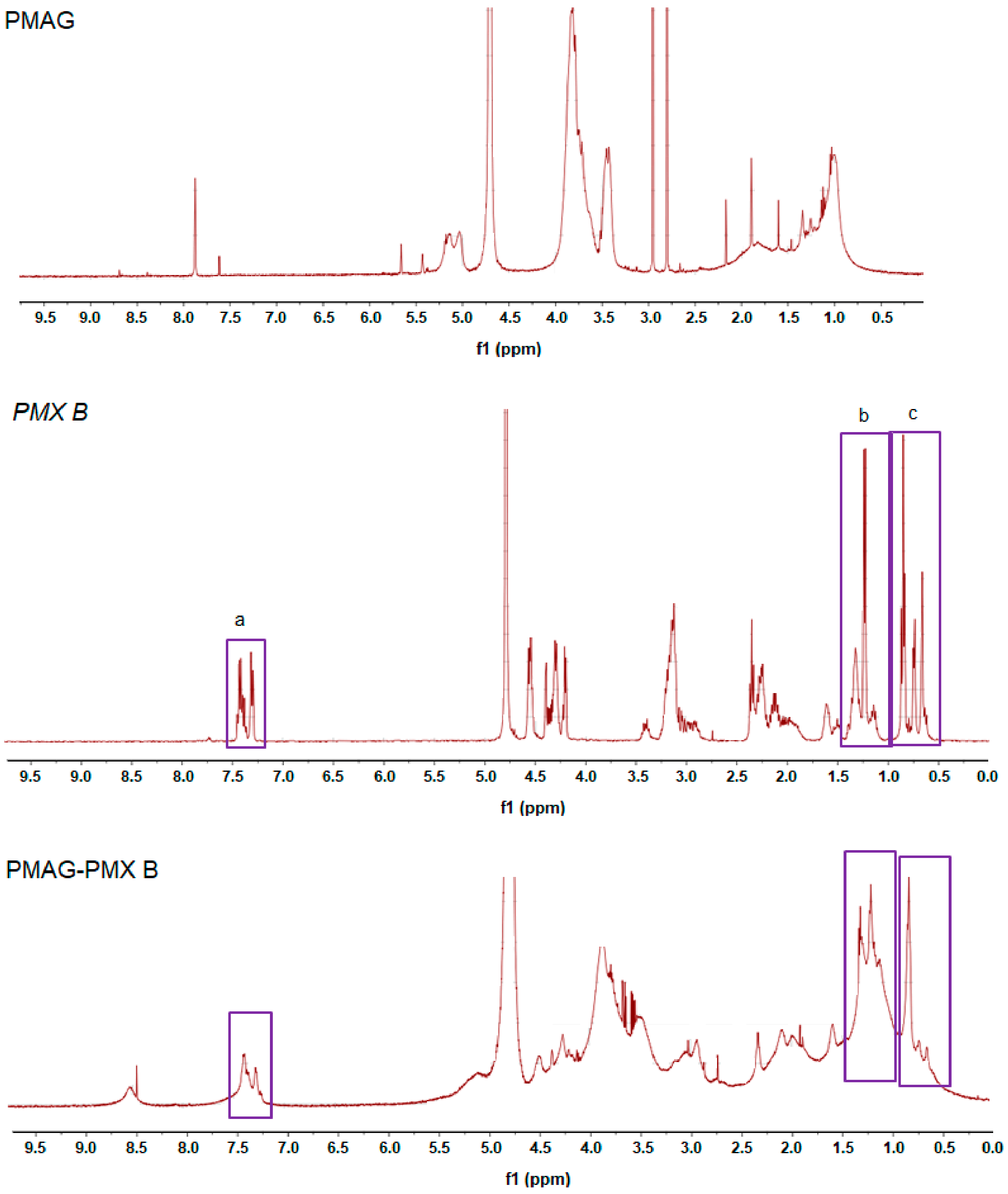

Figure S5. ^{1}H NMR spectra of PMX B, PMAG and PMAG-PMX B conjugate (D_2O, 25 °C). Signals in the boxes: *a* – aromatic protons from phenylalanine in PMX B; *b* and c – protons of terminal of $-CH_2$ and $-CH_3$ groups of PMX B cycle and aliphatic tail.
